# ABS-SmartComAgri: An Agent-Based Simulator of Smart Communication Protocols in Wireless Sensor Networks for Debugging in Precision Agriculture

**DOI:** 10.3390/s18040998

**Published:** 2018-03-27

**Authors:** Iván García-Magariño, Raquel Lacuesta, Jaime Lloret

**Affiliations:** 1 Department of Computer Science and Engineering of Systems, University of Zaragoza, 44003 Teruel, Spain; lacuesta@unizar.es; 2 Instituto de Investigación Sanitaria Aragón, University of Zaragoza, 50009 Zaragoza, Spain; 3 Integrated Management Coastal Research Institute, Universitat Politècnica de València, 46022 València, Spain; jlloret@dcom.upv.es

**Keywords:** agent-based simulation, smart communication protocols, agent-based social simulation, multi-agent system, agent-oriented software engineering, agriculture, sensor network

## Abstract

Smart communication protocols are becoming a key mechanism for improving communication performance in networks such as wireless sensor networks. However, the literature lacks mechanisms for simulating smart communication protocols in precision agriculture for decreasing production costs. In this context, the current work presents an agent-based simulator of smart communication protocols for efficiently managing pesticides. The simulator considers the needs of electric power, crop health, percentage of alive bugs and pesticide consumption. The current approach is illustrated with three different communication protocols respectively called (a) broadcast, (b) neighbor and (c) low-cost neighbor. The low-cost neighbor protocol obtained a statistically-significant reduction in the need of electric power over the neighbor protocol, with a very large difference according to the common interpretations about the Cohen’s d effect size. The presented simulator is called ABS-SmartComAgri and is freely distributed as open-source from a public research data repository. It ensures the reproducibility of experiments and allows other researchers to extend the current approach.

## 1. Introduction

Smart communication protocols have been widely used for making communications more efficient in several kinds of networks. For instance, Nair et al. [1] applied a smart protocol for improving communications in vehicular ad-hoc networks (VANETs). Their protocol was focused on the fragmentation of messages and their aggregation for reducing redundant data on transmissions. In addition, smart communication protocols have reduced the bandwidth of communications with 5G networks for monitoring chronic patients and warning of anomalous situations, when having large amounts of users (e.g., 1000 patients) [2]. The works about smart communication protocols have a wide range of purposes such as energy consumption reduction, balanced communications, interconnection of different device kinds and reduction of transmitted data. In addition, Khan and Faheem [3] presented a smart communication system for reducing energy losses in smart grids. Their approach showed its utility in a case study from Pakistan. They estimated that their approach could reduce the power theft from 25–5% in this country. The scan-based movement-assisted sensor deployment method (SMART) [4] proposed a smart communication approach for analyzing the performance of WSNs with mobile nodes. It was aimed at achieving efficient balanced networks minimizing the existence of holes. Their optimized version of SMART spatially distributed the sensor nodes with the minimum number of moves when compared with other similar popular algorithms at the time (i.e., Diff, Exch and Vor). Moreover, a smart communication architecture was proposed for interconnecting different devices and sensors [5]. This smart communication architecture was focused on ambient-assisted living of elderly people and the comfort of caregivers. The smart communication approach of Aazam and Huh [6] focused on reducing the amount of transferred data in the context of the clouds of things. In their approach, the devices of the Internet of Things (IoT) applied data trimming before sending the data to the cloud. Nevertheless, none of these approaches proposed simulating smart communication protocols considering the most common aspects of precision agriculture such as usage of pesticide, crop health and the existence of bugs.

Some smart communications protocols have been implemented with multi-agent systems (MASs), since their emergent behavior usually relies on social interactions among agents. The corresponding MAS applications can be classified in relation to the hierarchy level of the corresponding decision-making processes with the categories of (a) the operative or final execution of a process, (b) strategic and (c) tactical or directive. In the category of operative decisions, some MAS applications automated decisions for achieving several real-time actions. For instance, Li et al. [7] presented a smart agent communication mechanism for evaluating the reliability of integrated energy systems. In their approach, the system did not depend on any global information of the whole system. Instead, their smart communications relied on distributed information and a decentralized communication algorithm. Their proposed decentralized algorithm improved the efficiency with respect to a centralized solution. In particular, it reduced the system interruption frequency index by 5.1%, and it decreased the expected energy not supplied index by 9.8%. In addition, Bosse and Pantke [8] introduced a smart communication protocol for networks with error-prone nodes and with irregular densities of nodes over the space. Their approach was implemented with an MAS. In their approach, agents managed sensors and communicated among each other to transfer and share energy among neighbors when necessary. Agents had different energy need states for facilitating communication and coordination among neighbors. In the category of strategic decisions, some MAS applications implemented elaborated strategies for obtaining some emergent behaviors. For example, some communication protocols have been proposed for achieving agreement in MASs. In particular, the Delphi method has inspired smart communication protocols for reaching agreement in the assessment of documents. An MAS completely implemented this method [9]. It evaluated the relevance of documents, from several sets of documents. Each document set represented the knowledge of an expert in a particular science field. It improved the accuracy over the evaluations with traditional information retrieval techniques from each set of documents. Some works about MAS applications focused on the category of tactical or directive decisions. For instance, the measurement of agent communications can guide the definition of smart communication protocols. There is a metrics suite that measured communications of MASs and evaluated whether communications were properly balanced [10]. This metrics suite detected undesirable communication patterns. These metrics were also useful for assessing whether some smart communication protocols were malfunctioning. The existence of these works motivates the use of MASs for simulating smart communication protocols in the current work. However, none of these works implemented an MAS for simulating smart communication protocols in the specific context of precision agriculture. The current approach addresses this purpose with operative decision-making processes.

There are some agent-based simulators (ABSs) for simulating land use in relation with agriculture. For instance, the Spatially Explicit Agricultural Dynamics (SEAD) [11] software package simulated evolutions of agricultural land use by means of an agent-based model. The calibration of their system was based on remotely-sensed data. They detected agricultural lands by Landsat images. They related the agricultural spread to different factors such as water availability, soil type and road infrastructure. In this line of research, Ralha et al. [12] also proposed an ABS for simulating expansion and changes of land use. They documented their approach with the overview, design concepts and details (ODD) protocol. They also used Landsat images, and they experienced their approach with a case study from Brazilian Cerrado. Yang et al. [13] also used an ABS for simulating the use of croplands. They focused on modeling of human behaviors. In particular, they modeled and simulated behaviors of farmers and governments. They illustrated their approach with the reconstruction of about three centuries of Shandong province in China. The simulated reconstruction results were similar to the real ones considering the historical map and a point-by-point comparison. Zhang et al. [14] also presented an ABS for simulating land use with a case study in China. They considered agriculture as one of the main purposes of land use. The experimental results showed that their ABS was able to rationally allocate the quantitative structure of land use satisfying the entered constraints of multiple objectives such as maximizing economic, ecological and social benefits. Nonetheless, these approaches did not provide the possibility of defining and simulating different smart communication protocols. Another relevant difference is that these ABSs about land use explored long-term behaviors, while the current approach about precision agriculture addresses automation and operative decisions that are frequently taken in short-term intervals.

In precision agriculture, technology could guarantee not only attending the basic needs of plants, but also anticipating their specific needs given several environment variables. An open challenge is to accurately predict the potential yield losses in order to take proper actions [15] considering aspects such as pesticides, diseases and climate. In agriculture, wireless sensor networks (WSNs) allow collecting information in a distributed way and sharing it among neighbor sensors. Smart communication protocols could efficiently guide the spread and management of this information. The applications of WSNs to precision agriculture could be classified considering their purposes such as irrigation and pesticide distribution. In the context of irrigation, Maurya and Jain [16] proposed a WSN that used region-based clustering for efficiently covering a given area. They used a hybrid routing for transmitting the data to a base station. In the line of edge-computing and smart communication protocols, their approach only sent the data of sensors when this information was requested. In the context of pesticide distribution, Valente et al. [17] used a WSN for monitoring crop in vineyards in combination with an unmanned aerial vehicle (UAV). In this way, the UAV was able to reach the information collected by isolated sensor clusters. Their approach could prevent crop damages and reduce pesticide wastage by mainly focusing on areas with bugs. In the different categories of agriculture WSNs, the data acquisition are normally necessary. Camilli et al. [18] analyzed different mechanisms of using WSNs for data acquisition from agriculture fields. They discussed the benefits and drawbacks of their distributed approach in comparison to a centralized approach. Their estimations reduced accuracy by only 3% when solely using local data. In contrast to all these works, the novelty of the current work relies on the support for developing and evaluating low-cost strategies in terms of energy and pesticide consumption that effectively avoid damages caused by bugs. For example, developers can assess different strategies that only perform communications among neighbor sensors.

Several works have aimed at reducing the energy consumption of WSNs in the context of precision agriculture. Jawad et al. [19] reviewed the works about energy-efficient WSNs for precision agriculture. They compared the different wireless technologies or protocols to determine which were the most efficient for precision agriculture. They found that ZigBee and LoRa were more efficient than other technologies such as WiFi or Bluetooth. They also discussed other challenges such as the ones related to heterogeneous sensors, fault tolerance, delay tolerance, battery life and security. In addition, Mirhosseini et al. [20] presented an improved binary quantum-inspired gravitational search algorithm (BQIGSA) for reducing energy consumption in precision agriculture WSNs. Their approach extended the life span of networks in comparison to other similar well-known algorithms. In particular, their algorithm improved the binary genetic algorithm (BGA), the binary particle swarm optimization (BPSO) and the quadrivalent quantum-inspired gravitational search algorithm (QQIGSA), obtaining a minimum reduction of 10.6% of energy consumption over each of these algorithms. The approach of Maurya and Jain [16] is also aimed at reducing the energy consumption of WSNs in agriculture. In particular, they used a fuzzy logic technique with a static and hybrid routing for reducing energy consumption in every data transmission. Furthermore, Ferrández-Pastor et al. [21] presented a ubiquitous sensor network platform in precision agriculture. Their platform used edge computing in IoT devices adding a computing layer in the local area. They assessed their platform by developing a greenhouse with hydroponic crop production. Their approach reduced water consumption by 20% in comparison to the consumption with soil.

On the whole, to the best of the authors’ knowledge, the literature lacks an appropriate simulator that allows one to define and simulate different smart communication protocols in precision agriculture with a WSN, specifically for pesticide distribution, among other purposes. A simulator of this kind could be useful for achieving an appropriate balance among communications with a low need of electric power, reduction of pesticide wastage and preservation of crop health. The system could coordinate fumigating infected areas and neighboring ones for avoiding the spread of bugs. Smart communication protocols could be useful for managing communications in an efficient way, avoiding redundant transmission of data, which could reduce the necessary electric power in the corresponding WSNs.

The current work addresses the problem of achieving the right balance among the needs of electric power, the avoidance of bugs and the reduction of pesticide wastage. A subsequent problem is the lack of simulation tools for testing smart communication protocols in this context. Notice that smart communication protocols could access local information with negligible electric power in comparison to the one needed for two sensors via wireless communication. Sensors may use batteries instead of wired electricity, and the electric power and the corresponding energy consumption would be really important in this scenario.

In this context, the current work presents a novel open-source ABS of smart communication protocols for debugging in precision agriculture called ABS-SmartComAgri. The next section presents this simulator as the main material in this research work.

## 2. Materials and Methods

### 2.1. ABS-SmartComAgri

ABS-SmartComAgri simulates a WSN that detects bugs and debugs a field with precision agriculture. The main goal of ABS-SmartComAgri is to allow engineers to define and simulate smart communication protocols in this context. In the current work, the main goal of smart communication protocols is to reduce the necessary electric power and its corresponding energy consumption, as well as to keep the crop healthy without excessively fumigating it.

ABS-SmartComAgri is freely distributed as open-source from a public dataset in the Mendeley research data repository (http://dx.doi.org/10.17632/yzmt73x8j8.1). This not only guarantees the reproducibility of experiments, but also allows other researchers and engineers to extend the underlying simulation framework or reuse any of its components. ABS-SmartComAgri has been developed following the process for developing efficient ABSs (PEABS) [22]. In particular, we have implemented this simulator with a Unity 3D engine and the C# object-oriented programming language.

#### 2.1.1. Agent-Based Framework for Simulating Debugging in Precision Agriculture

ABS-SmartComAgri is based on a novel agent-based framework for simulating different strategies in the context of smart communication protocols in agriculture. The agent architecture of the current approach includes the following agent types:

- Sensor agent: This agent has the possibility of detecting bugs and fumigating a crop area. In real world, this detection would be accomplished with computer vision (CV) techniques as commonly done in the precision agriculture literature [23]. This agent can communicate with other sensor agents to warn them of the existence of bugs. This agent also decides when to fumigate. Thus, this agent would need to be connected to an actuator system of precision agriculture. The use of electric power is simulated considering the communications among these agents.
- Bug agent: This agent represents a bug in a field. It eats the crop if this has not been previously fumigated. It randomly moves in different directions. It dies when exposed to pesticide for a few hours.
- Crop agent: This represents the crop that lives and grows in a specific area. Bugs can eat the corresponding crop. This agent determines crop health as a percentage.
- Agriculture observer agent: This agent tracks the activity of the ABS in order to report its evolution and its final state.

The simulation starts with a grid of sensor agents with the dimension indicated by the user. Each of these sensors monitors a specific area. The simulation starts with the initial number of areas with bugs indicated by a parameter. In addition, some bugs start randomly at some timeline points in some random locations. In each simulated iteration (i.e., hour), the system makes a nondeterministic decision about whether to simulate the appearance of new bugs. This decision is implemented following TABSAOND (the technique for developing ABS apps and online tools with nondeterministic decisions) [24]. This decision is simulated with the following formula that determines whether to simulate the appearance of new bugs and their quantity:

(1) $$d_{b}=\left\{\begin{array}{cc} r_{b}, & \mathrm{if}\;r_{a}\leq p_{a}\\ 0, & \mathrm{otherwise} \end{array}\right.$$

where $d_{b}$ is the number of bugs that appear in the field, $p_{a}$ is a parametric probability about whether bugs appear in the field, $r_{a}$ is a random number in the [0,1) interval for simulating this probability and $r_{b}$ is a random number between zero and the parametric maximum number of bugs $M_{b}$.

If a bug remains exposed to pesticides for a certain period (measured in the number of simulated iterations), it dies. Each bug agent has a health indicator variable that decreases when staying in an area that has been fumigated. It can eat any crop that has not been fumigated previously (i.e., in the previous iteration). Each bug can randomly fly from one zone to the neighbor zones or stay in the same one. The current bug location is calculated from its previous one with the following equation:

(2) $$\vec{P_{i,t}}=\vec{(Clamp\left(P_{i,t-1}.x+r_{x},0,N\right),Clamp\left(P_{i,t-1}.y+r_{y},0,M\right)}$$

where $P_{i,t}$ is a vector that determines its zone within the grid for a given *t* time (also applied to the previous iteration as t−1), Clamp(v,min,max) is a function that clamps the *v* value between the min and max values, V.x and V.y represent respectively the first and second coordinates of any *V* vector and $r_{x}$ and $r_{y}$ are two different integer random values in the [−1,1] interval. Notice that the actual *P* position of a bug is selected as any random position within the corresponding zone of the grid determined by the indices of $P_{i,t}$.

Each sensor agent is also responsible for deciding when to fumigate. It can sense bugs in their zone. It can also communicate with any other sensor agent by indicating its position in the grid. It includes specific methods for sending messages to all the sensors (referred to as “broadcast”) and for sending messages to their neighbor sensor agents (denoted as “send neighbors”). Developers can define smart communication protocols by indicating when each sensor communicates with other sensors and when it fumigates. Protocols can use all the local information and that previously received by other sensors. Notice that each sensor can store information for later applying any learning process aiming at reducing the necessary electric power and pesticide consumption. Section 2.2 further describes how to define these smart protocols introducing the ones used in the experimentation. Each sensor records the power used when sending messages to other sensors. In this way, the average power can be measured to assess each smart communication protocol.

If a bug eats crop in a zone, then the health indicator of the corresponding crop agent decreases. The crop grows in each iteration repairing possible damages of bugs. However, bugs eat much faster than the crop grows.

In the current approach, the following features can be tracked during and after simulations:

- Average power: This measures the average electric power used by each active station with a sensor (i.e., either communicating or fumigating), computed on an hourly basis. This feature allows practitioners to compare communication protocols about energy consumption.
- Crop health: This indicator shows the percentage of crop that has not been eaten by bugs. For example, a zone has 100% crop health if the zone has never hosted bugs. When a zone has bugs, then these eat the crop, gradually reducing this indicator. This feature can be used as an indicator of how well the precision agriculture system reacts to bug attacks. The system calculates an average of this feature for all the zones to provide a global measure about the efficacy of a smart communication protocol in protecting the crop from bugs.
- Percentage of alive bugs: This represents the number of alive bugs divided by the total number of bugs regardless if these are alive or dead.
- Pesticide quantity: This measures the average pesticide quantity consumed per sensor from the simulation beginning. The crop should not be excessively and unnecessarily exposed to pesticides in order to obtain a healthy product. In addition, less money is spent when using low amounts of pesticide.

#### 2.1.2. User Interface

**Figure 1:**
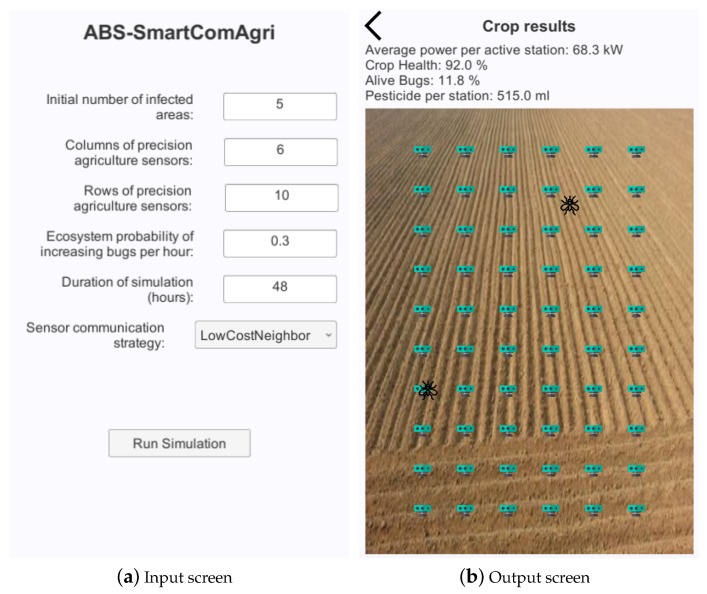
User interface (UI) of ABS-SmartComAgri.

Figure 1 shows the user interface (UI) of ABS-SmartComAgri. Figure 1a presents the input screen for entering input parameters. Users can indicate the initial number of zones with bugs. The other two parameters determine the numbers of columns and rows of the sensor grid. Users can also configure the probability with which new bugs appear in each hour. The corresponding bugs are assumed to fly from outside the field or be born inside it. Another input parameter allows users to set the simulated time by indicating the number of hours. Finally, users can select a smart communication protocol strategy from a drop-down menu.

Figure 1b shows the screen that presents the simulation results. More concretely, it shows a graphical representation of the crop field with the sensors of the WSN. The presence of bugs is represented graphically with some simple images in the corresponding locations of the map. The simulator provides three global measures that can assess smart communication protocols in precision agriculture. The first measure determines the average power that each active station consumes in kW computed on an hourly basis. Notice that sensors are normally asleep when there is not any bug risk of having it. It also measures crop health, which implicitly determines the amount of crop that has been eaten by bugs. It shows the percentage of alive bugs divided by all the bugs including dead and alive ones. Finally, it indicates the amount of used pesticide in milliliters (mL).

In simulation evolutions, all these measures are stored in each iteration in an external file, so that these evolutions can be analyzed and studied.

### 2.2. Definition of Debugging Strategies with Smart Communication Protocols

**Figure 2:**
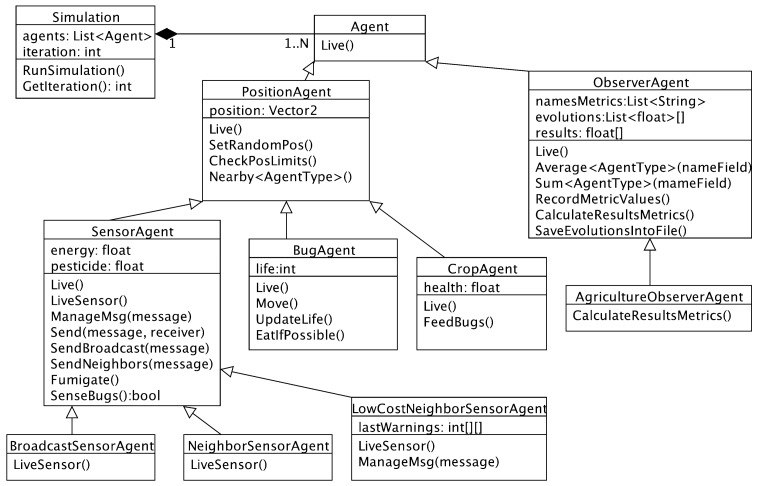
Class diagram of the excerpt of ABS-SmartComAgri concerning its agent types.

ABS-SmartComAgri supports the definition of debugging strategies with smart communication protocols in precision agriculture by means of an underlying framework. This framework uses PEABS for defining agent types, which are represented as object-oriented classes. Figure 2 presents the class diagram about the agent types of ABS-SmartComAgri. It includes the definitions of the debugging strategies presented in this section.

It is worth noting that some agent types extend some generic agent types that have some common operations. For example, sensor agents, bug agents and crop agents are located in a specific position of the simulated map. Thus, all these agent types extend the position agents that have operations related to positions. In addition, the agriculture observer agent is implemented as an extension of a generic observer agent. This agent has operations such as calculating average properties of other agent types, recording the values of certain metrics and saving the evolutions of these values into a file.

Debugging strategies can be defined by extending the sensor agent and implementing its “live sensor” and “manage Msg” methods. The live sensor method is called once every iteration and allows strategies to define periodical actions. Normally, this method is useful for sensing bugs and taking proper actions. Each sensor agent can decide (a) whether to fumigate or not, (b) which messages to send if any and (c) to which sensor agents send these.

The purpose of the manage Msg method is to define reactive behaviors of sensor agents when receiving messages. Each message is received by a method parameter. This method is only executed when another sensor agent sends a message to the current one.

Strategies can use inherited methods of the sensor agent. Any of the two aforementioned overridden methods can invoke the “fumigate” method for fumigating. Once an agent calls this method, the sensor is assumed to be fumigating for one iteration (i.e., one simulated hour). If a sensor is fumigating in one iteration, it fumigates the same amount of pesticide regardless if the method is called once or more times in this iteration. This simplifies the implementation of strategies, as these do not need to check whether a sensor is already fumigating.

In the definition of strategies, the developer can use the “sense bugs” method for checking whether a sensor is sensing bugs. This method returns a Boolean value that determines whether the sensor detects alive bugs.

Sensor agents can communicate with each other by sending messages. In particular, a strategy can invoke the “send” method for sending messages to any other agent. This method receives two parameters respectively for determining (a) the target sensor agent by its position in the sensor grid and (b) the message. The latter is represented with an enumeration type, to indicate different possible messages. For now, all the proposed smart communication protocols use the same message named “warn bugs”, which alerts about the existence of alive bugs. The send method automatically adds more information to any message, including (1) the sender identified by its grid position and (2) the simulation timeline spot represented with the simulation iteration (i.e., the number of elapsed simulated hours). The invocation of the send method automatically tracks average power used by communications. The two methods “broadcast” and “send neighbors” implicitly send a message to a group of sensors. This group is all the sensors or only the neighbor sensors, respectively for the two methods.

The implementations of the agent methods can invoke any method from the PEABS framework such as the “get iteration” method to know the timeline spot determined by the simulation iteration.

Smart communication protocols can use all the necessary data structures, by including fields in the C# classes of strategy definitions. These structures can be used for learning information and avoiding the transmission of redundant data.

The current approach is illustrated with three different precision agriculture strategies with their corresponding underlying communication protocols.

#### 2.2.1. Broadcast Strategy

The “broadcast” strategy simply uses the WSN for fumigating all the field when any sensor detects an area with bugs. The purpose of this strategy is to illustrate the current approach with a straightforward communication protocol. This strategy is defined with two rules. If a sensor detects a bug, it fumigates and warns all the other sensors of the existence of bugs. If a sensor is warned, it fumigates. These two rules were respectively incorporated by implementing the live sensor and manage Msg methods. The behavior of sensors is formalized with the following equation:

(3) $$\forall s_{x,y}\in S:SenseBugs\left(s_{x,y},t\right)\Rightarrow Fumigate\left(s_{x,y},t\right)\wedge Broadcast\left(s_{x,y},WarnBugs,t\right)$$

where *S* is the set of sensors, $s_{x,y}$ denotes the sensor located in the grid position $\vec{\left(x,y\right)}$ in this equation and the next ones, $SenseBugs(s_{x,y},t)$ determines whether the sensor $s_{x,y}$ has detected any bug in its area, *t* represents the timeline spot in this equation and the following ones and $Fumigate(s_{x,y},t)$ represents that the corresponding sensor fumigates in the area of $\vec{\left(x,y\right)}$ position in time *t*. WarnBugs represents a warning of the existence of bugs in this equation and some of the next ones. $Broadcast(s_{x,y},t)$ represents a broadcast from a sensor to all the others and is formally defined with the following equation:

(4) $$\begin{array}{c} Broadcast\left(s_{x,y},WarnBugs,t\right)\Rightarrow \forall i\in \left[0,M\right),j\in \left[0,N\right):\\ \left(i\neq x\wedge j\neq y\right)\Rightarrow Send\left(s_{x,y},s_{i,j},WarnBugs,t\right) \end{array}$$

where *M* and *N* are the two dimensions of the sensor grid; and Send(s,r,m,t) represents an actual communication of an *m* message, from a sender sensor *s* to a receiver sensor *r* in time *t*.

In the manage Msg method, the management of the reception of messages is also formally defined with the Send predicate, by defining actions in the receiver. In this particular strategy, reception of messages is defined as follows:

(5) $$Send\left(s_{x,y},s_{i,j},WarnBugs,t\right)\Rightarrow Fumigate\left(s_{i,j},t\right)$$

where $Fumigate(s_{i,j},t)$ represents that $s_{i,j}$ sensor fumigates in the area of the $\vec{\left(i,j\right)}$ grid position during the *t*-th hour of the simulated time.

#### 2.2.2. Smart Neighbor Strategy

**Figure 3:**
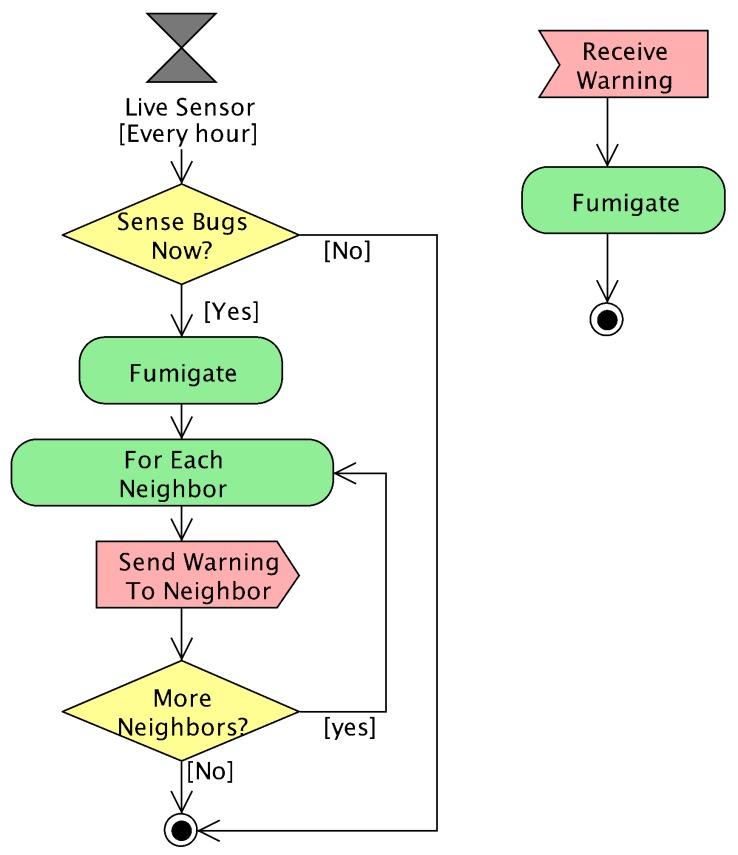
Functional block diagram of the smart neighbor strategy.

The smart “neighbor” strategy is based on communicating warnings of bugs only between neighbor areas. Figure 3 shows the functional block diagram of this communication protocol. This strategy uses context awareness, since each sensor is aware of its location. It is also aware of its neighbor sensors. It communicates with them to maintain coordination for effectively debugging in precision agriculture. In this way, the multi-agent-based coordination of this strategy achieves an emergent smart behavior. Sensor agents are proactive and take the initiative every simulated hour by means of the periodical event referred to as “live sensor”. In some cases, the current sensor sends messages to peer neighbor sensors. Sensor agents are also reactive. When a sensor agent receives a message (treated as an event), it takes some actions.

This strategy relies on the assumption that bugs only move to neighbor areas in each short time interval. In this way, if a sensor detects any bug, it fumigates its area and warns its neighbor sensors. If a sensor is warned by another agent, it fumigates. Hence, we formalized the behavior of the live sensor method with the following equation:

(6) $$\forall s_{x,y}\in S:SenseBugs\left(s_{x,y},t\right)\Rightarrow Fumigate\left(s_{x,y},t\right)\wedge SendNeighbors\left(s_{x,y},WarnBugs,t\right)$$

where $s_{x,y}$, SenseBugs and Fumigate have the same behavior as in the previous strategy. The communication to neighbors $SendNeighbors(s_{x,y},t)$ is formalized with the following equation:

(7) $$\begin{array}{c} SendNeighbors\left(s_{x,y},WarnBugs,t\right)\Rightarrow \forall i\in \left[x-1,x+1\right],j\in \left[y-1,y+1\right]:(i\neq x\wedge j\neq y\wedge \\ i\in \left[0,M\right)\wedge j\in \left[0,N\right))\Rightarrow Send\left(s_{x,y},s_{i,j},WarnBugs,t\right) \end{array}$$

The management of the reception of warning messages is the same as in the previous strategy. The corresponding sensor fumigates when receiving any warning message. The formalization of the manage Msg method is the one previously introduced in Equation (5).

#### 2.2.3. Smart Low-Cost Neighbor Strategy: With a Reduced Number of Communications

**Figure 4:**
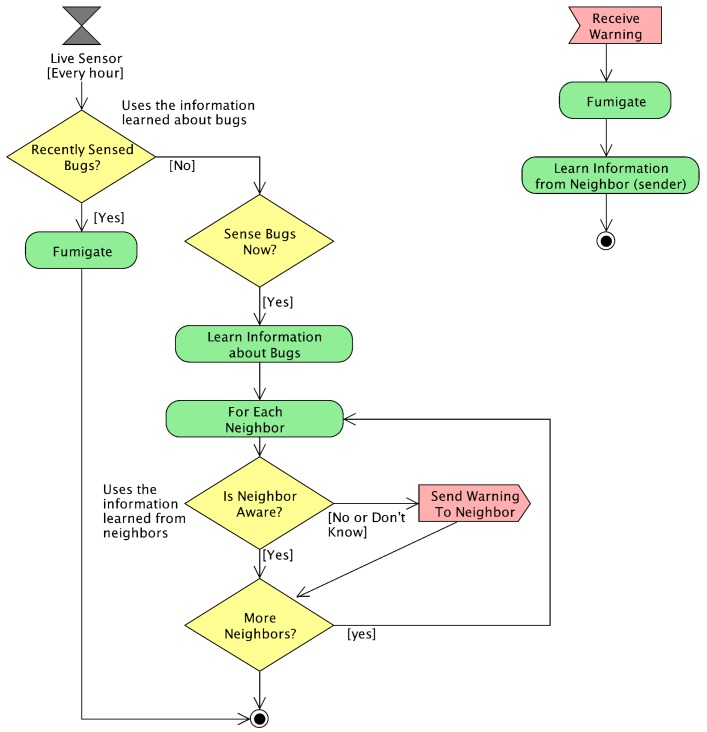
Functional block diagram of the smart low-cost neighbor strategy.

The “low-cost neighbor” strategy uses a smart communication protocol that learns from past communications in order to avoid redundant messages. Figure 4 shows the functional block diagram of this smart communication protocol. This strategy also uses proactive and reactive agents. It also uses context-awareness information for coordination among neighbors. The main improvement of this strategy is its ability to learn from the interactions with peers to avoid redundant communications. More concretely, each sensor recalculates its context-aware information for effectively predicting the states of its neighbors. When receiving a warning message from another sensor, it learns that this sender sensor has detected bugs to avoid warning it back. Each sensor also learns from the existence of bugs to keep fumigating for more than one simulated hour. In this way, the whole system saves energy globally, since the current sensor does not need to be warned back, even if the bugs move to a neighbor area. In this way, the current area is safely fumigated in case the bugs come back to this area.

In this strategy, each sensor stores information about the last time it has received any warning from any of its neighbors. In particular, each sensor stores this information in a 3 × 3 matrix called “last warnings” and referred to as *W*:

(8) $$W\left(s_{x,y}\right)=\left[\begin{array}{ccc} w_{0,0} & w_{0,1} & w_{0,2}\\ w_{1,0} & w_{1,1} & w_{1,2}\\ w_{2,0} & w_{2,1} & w_{2,2} \end{array}\right]$$

where $s_{x,y}$ is the current sensor, $w_{1,1}$ refers to this sensor and each other $w_{i,j}$ represents the neighbor sensor in the corresponding local position with respect to $w_{1,1}$. Each $w_{i,j}$ contains the last time *t* in which this neighbor sent a warning. Each $w_{i,j}$ of a sensor $s_{x,y}$ is also denoted as $W\left(s_{x,y}\right)\left[\vec{\left(i,j\right)}\right]$ in some of the next equations.

When a sensor agent receives a message, it fumigates and updates the last warning matrix to properly store the information about the last warnings. The manage Msg method is implemented with the following equation:

(9) $$Send\left(s_{x,y},s_{i,j},WarnBugs,t\right)\Rightarrow Fumigate\left(s_{i,j},t\right)\wedge UpdateLastWarning\left(s_{x,y},s_{i,j},t\right)$$

where Send, WarnBugs, *t* and Fumigate have the same behavior as in the previous equations, $s_{x,y}$ is the sender sensor and $s_{i,j}$ is the receiver one. UpdateLastWarning has the following definition for updating the *W* matrix:

(10) $$UpdateLastWarning\left(s_{x,y},s_{i,j},t\right)\Rightarrow W\left(s_{i,j}\right)\left[GlobalToLocal\left(\vec{\left(x,y\right)},\vec{\left(i,j\right)}\right)\right]=t$$

where $GlobalToLocal(\vec{G},\vec{R})$ transforms the $\vec{G}$ global position of a sensor into the local position in the *W* matrix. This is obtained with respect to the $\vec{R}$ reference position with the following equation:

(11) $$GlobalToLocal\left(\vec{G},\vec{R}\right)=\left(\vec{G}-\vec{R}\right)+\vec{\left(1,1\right)}$$

The local position is calculated as the target position minus the reference position. Notice that this approach sums the $\vec{\left(1,1\right)}$ vector to adapt its position in the *W* matrix with respect to the $w_{1,1}$ position of the matrix.

In this strategy, when each sensor senses a bug, it fumigates for a duration longer than just one hour. This fumigation duration is represented with the *d* internal parameter of the strategy using hours as the unit. The $b(s_{x,y})$ variable represents the last time the current sensor detected a bug. Moreover, when the sensor detects a bug, it warns its neighbor in a smart way, avoiding redundant communications. In particular, it avoids sending warnings to the neighbors that sent warnings in the last *d* hours. The live sensor method is formalized as follows:

(12) $$\begin{array}{c} LiveSensor\left(s_{x,y},t\right)\Rightarrow \left(\left(\left(t-b\left(s_{x,y}\right)\right)\leq d\right)\to Fumigate\left(s_{x,y},t\right)\right)\wedge \\ (((\left(SenseBugs\left(s_{x,y},t\right)\wedge \left(t-b\left(s_{x,y}\right)\right)>d\right)\to (SendNeighborsSmartly\left(\left(s_{x,y},s_{i,j},WarnBugs,t\right)\right)\wedge \\ Assign(b\left(s_{x,y}\right),t))) \end{array}$$

where LiveSensor represents the periodically-invoked method of each sensor agent, SendNeighborsSmartly represents the smart communication avoiding redundant messages and Assign(b,t) represents an assignment of the current time to the variable *b* about the last detected bugs. All the other variables have the meanings previously introduced.

The smart communication SendNeighborsSmartly avoids sending the warnings to the neighbors that sent warnings in the last *d* hours and consequently are still fumigating. This is represented with the following equation:

(13) $$\begin{array}{c} SendNeighborsSmartly\left(s_{x,y},t\right)\Rightarrow (\forall i\in \left[x-1,x+1\right],\forall j\in \left[y-1,y+1\right]:\\ (i\neq x\wedge j\neq y\wedge i\in \left[0,M\right)\wedge j\in \left[0,N\right)\wedge \left(\left(t-W\left(s_{x,y}\right)\left[\vec{\left(i,j\right)}\right]\right)>d\right)\\ \to Send(s_{x,y},s\left[LocalToGlobal\left(\vec{\left(x,y\right)},\vec{\left(i,j\right)}\right)\right],WarnBugs,t)) \end{array}$$

where $LocalToGlobal(\vec{R},\vec{L})$ obtains the global position of the sensor at a local position in the *W* matrix, with the following formula:

(14) $$LocalToGlobal\left(\vec{R},\vec{L}\right)=\left(\vec{L}-\vec{\left(1,1\right)}\right)+\vec{R}$$

where $\vec{L}$ is the local position of the neighbor sensor in the *W* matrix and $\vec{R}$ is the reference position of the current sensor.

### 2.3. Method for Evaluating the Current Approach

In order to assess the novel ABS-SmartComAgri simulator, we executed the three debugging strategies with their smart communication protocols. In this way, we can evaluate both the novel simulator tool and the strategies. All the strategies were executed with the same simulation scenario for obtaining a fair comparison.

The current approach was simulated in a field of 1.0 × 1.6 km. In this simulation scenario, there was a grid of 10 × 16 of sensors. We denote each 100 × 100 m space monitored by each sensor as an area. We established that two areas initially contained bugs. We simulated the appearance of new bugs with a probability of 0.10 per hour. Bugs moved around when alive. We performed simulations of 48 h.

We executed each communication protocol 100 times for avoiding bias due to the nondeterministic behavior of the simulator. In particular, Table 1 indicates the input parameter values used in all the experiments.

Firstly, we compared the final simulation results of the three strategies considering the averages of all the simulations and the standard deviations (SD).

We performed several statistical tests for assessing the significance of some differences. Since the broadcast strategy is too basic and uses an excessive amount of pesticide, we discarded this strategy in some of these tests. In addition, we calculated the effect sizes for measuring the differences between neighbor and low-cost neighbor strategies.

The current work also compared the simulation evolutions of the features with graphs. We analyzed these graphs based on our observation. This work focused on comparing the simulation evolutions of the neighbor and low-cost neighbor communication protocols, since these two were the ones that obtained the best results. The broadcast strategy was omitted in this detailed comparison due to its basic communication behavior and its overuse of pesticide.

## 3. Results

**Figure 5:**
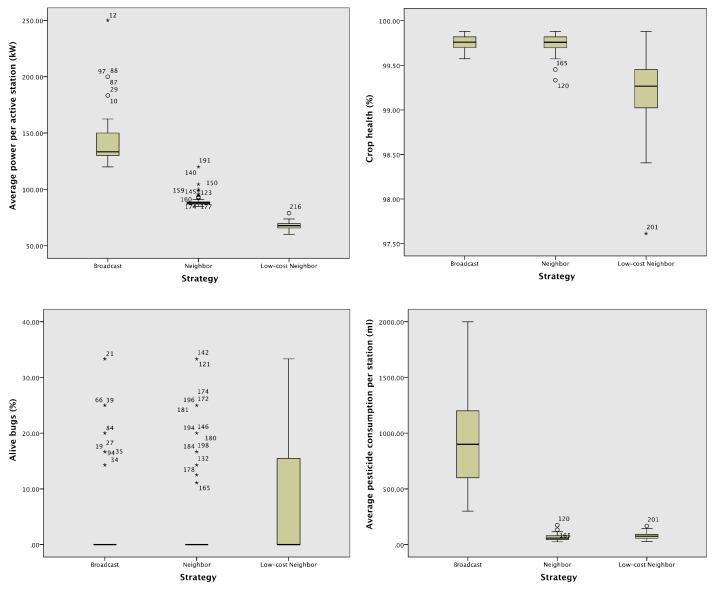
Boxplots for comparing the three communication protocol strategies.

Table 2 presents the results of the smart protocols by showing the means and the SD between parentheses of (a) the average power per active station, (b) the crop health represented as a percentage, (c) the percentage of alive bugs and (d) the average pesticide consumed by each station. Figure 5 graphically compares these means with boxplots.

We applied Levene’s *t*-test to assess the homogeneity of variances before selecting the statistical test for comparing the means. Since this test failed with a significance below the 0.001 level, we discarded it using the one-way ANOVA test. We applied Welch’s t-test and the Brown–Forsythe test, because these tests are robust for comparing quantitative independent samples even when variances are unequal. Table 3 shows the results of comparing the three communication protocol strategies. All the analyzed metrics obtained significant differences among the three strategies. These differences were significant with a level of 0.001 in all the metrics, except the percentage of alive bugs, for which the level was 0.01.

Table 4 shows the results of Welch’s t-test and the Brown–Forsythe test comparing the neighbor and the low-cost neighbor strategies. The differences in the percentage of alive bugs became non-significant. The differences in the power and crop health remained very significant. The difference of pesticide consumption reduced its significance, increasing its *p*-value.

In order to measure the differences between these two strategies, Table 5 shows the mean differences and the Cohen’s d effect sizes. According to the these effect sizes, the greatest difference between the two strategies was the reduction of power of the low-cost neighbor strategy over the neighbor strategy. The Cohen’s d of this difference was −5.75, which is very large according to the interpretations of Rosenthal [25]. The low-cost neighbor strategy used slightly more pesticide with a small-medium effect size according to the guidelines of Cohen [26]. The effect size of alive bugs was small according to the same guidelines.

**Figure 6:**
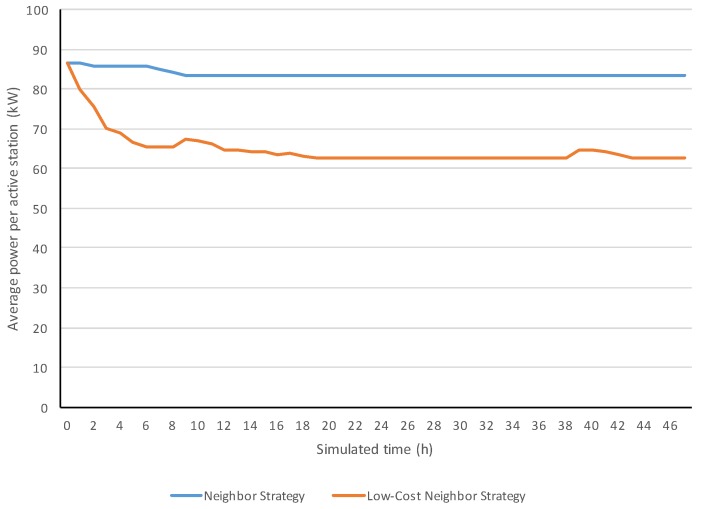
Simulation evolutions of the average power per active station.

**Figure 7:**
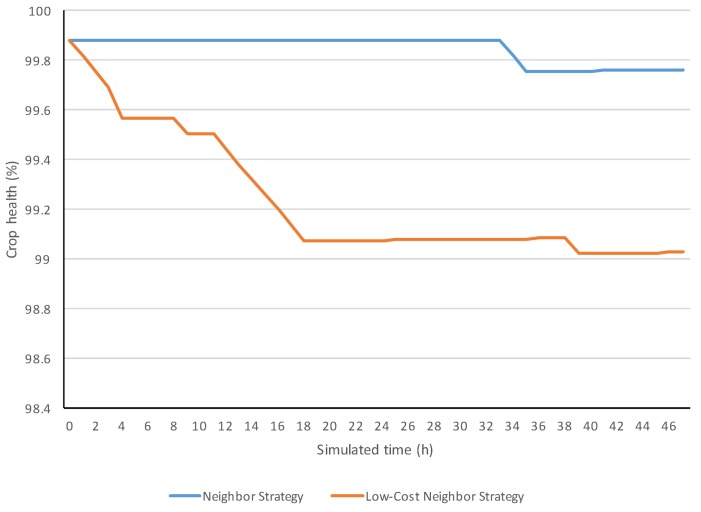
Simulation evolutions of the crop health.

Figure 6 compares two simulation evolutions of power from respectively the neighbor and low-cost neighbor strategies. Both strategies started with a similar power (around 87 kW). However, the low-cost neighbor strategy decreased its power, reaching 63 kW, while the neighbor strategy maintained its power around 83 kW. Figure 7 shows the evolution of the crop health for the two analyzed strategies.

**Figure 8:**
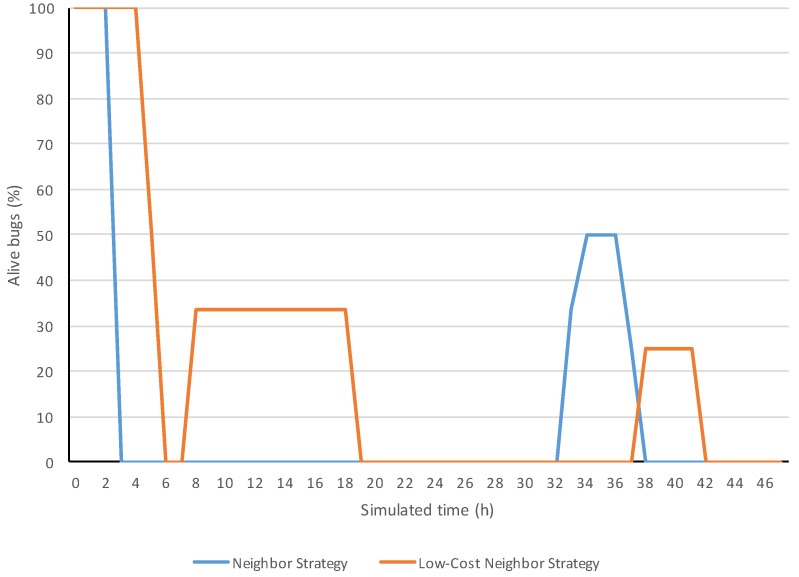
Simulation evolutions of the percentage of alive bugs.

**Figure 9:**
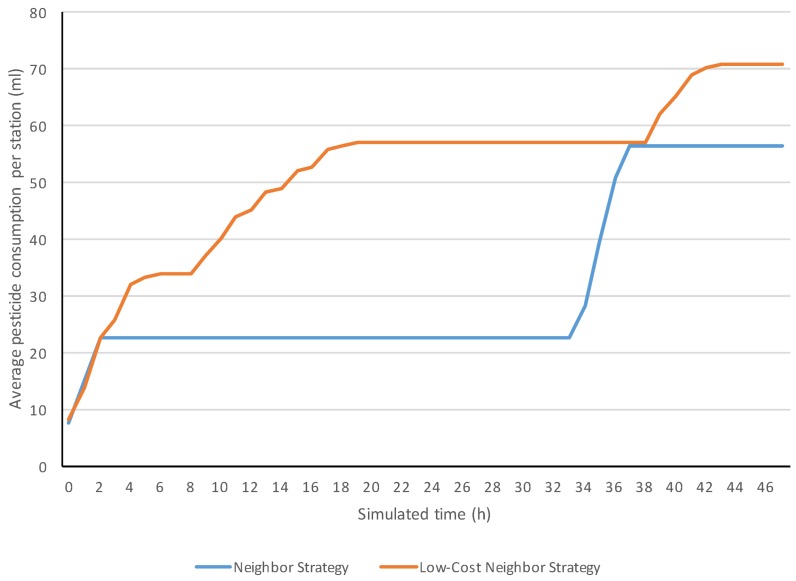
Simulation evolutions of average pesticide consumption per station.

In Figure 8, the evolution examples of alive bugs show that both strategies were able to exterminate bugs. The bugs of the two initially infected areas were exterminated in both strategies since the alive bugs dropped from 100–0% at the beginning. The increases of alive bugs represented that some bugs entered in the field or were born inside it. One can notice these appearances by the step increases from 0% to any value. Figure 9 shows examples of the evolutions of the pesticide consumed from the beginning.

## 4. Discussion

One can observe that the lowest necessary electric power is achieved by the low-cost neighbor strategy. The reduction of necessary electric power was the main goal when designing the communication protocol. Thus, this smart protocol is the one that obtained the best results in this main goal, probably thanks to the learning process.

It is worth noting that the three protocols properly defended the crop as its health was above 99% with all the strategies. The high SDs of the final percentage of alive bugs (i.e., the SD was above 1.5-times the means in all cases) revealed that the final value of this metric was very variable. Notice that this metric mainly depended on the last simulated hours. Although all the strategies properly debugged any new bug, the final results probably mainly depended on whether the new bugs appeared in the last simulated hours.

Regarding the pesticide, the broadcast strategy unnecessarily overused pesticide, as it normally fumigated in all the sensors even if bugs were specifically located in a small area. The consumed pesticide is over ten-times more than any of the other two strategies. This was inappropriate as such overuse of pesticide could cause health problems for consumers. This also represented an unnecessary wastage of pesticide.

Although the effect size of crop health was very large, the absolute mean differences were small (i.e., about only 0.5%), and both neighbor and low-cost neighbor strategies obtained appropriate values (i.e., above 99%).

The evolutions of power showed that the learning process of the smart protocol of the low-cost neighbor allows reducing the power needed by avoiding some redundant communications. In fact, the first simulation iterations obtained similar values, but the low-cost neighbor gradually reduced its power until arriving at a stable average value.

It is worth highlighting that both neighbor and low-cost neighbor strategies kept crop health above 99% during all the evolution. Thus, these two strategies were appropriate regarding this matter.

As one can observe in the evolutions of alive bugs, both neighbor and low-cost neighbor strategies achieved extermination of the bugs, since their percentage of alive ones dropped to 0% for all the appearances of bugs.

The consumed pesticide is an accumulative measure, and consequently, it cannot decrease. The increasing parts of the evolutions correspond to the periods where the system was fumigating. One can observe that the fumigation periods matched the ones in which some bugs were alive according to the other evolution graph, since both evolution graphs were extracted from the same simulation.

In conclusion, both neighbor and low-cost neighbor strategies were appropriate considering crop health and alive bugs. The advantage of the low-cost neighbor strategy is the reduction of necessary electric power over the neighbor strategy.

## 5. Conclusions

The current work has presented a novel open-source ABS of smart communication protocols for debugging in precision agriculture with WSNs. This ABS has allowed defining and simulating three different communication protocols with statistically-significant differences among these mainly in necessary electric power, crop health and pesticide consumption. The usage of this ABS can be useful for prototyping and testing communication protocols in precision agriculture before actually deploying these with real WSNs.

A limitation of the current approach is the separation between the theoretical simulated model and reality. For example, sensor detection of bugs is assumed to function perfectly, but in practice, these sensors may not detect some kind of bugs in some kinds of situations. In addition, some kinds of bugs may not follow exactly random movement patterns. For example, some bugs fly in groups. In addition, the probability of appearing bugs may vary regarding the weather. In order to make more realistic models, sensors will use different detection probabilities for simulating error rates. To distinguish different kinds of bugs, we may define more complex bug behaviors. For instance, they could fly to the areas with higher amounts of crop (e.g., areas where other bugs have not eaten crop yet).

The current work is planned to be extended considering more aspects of precision agriculture with smart protocols. For instance, it could take different kinds of vegetables and fruits into account with their corresponding vulnerabilities. The simulator could also simulate several kinds of bugs with different repercussions in the corresponding kinds of vegetables and fruits. The ABS could also simulate the coordination between the precision agriculture system and the farmer so that the latter could just perform only the necessary agricultural activities. The estimation of necessary electric power for each communication protocol could be improved by considering the distance between sender and receiver sensors. This estimation could also consider possible obstacles such as trees and cottages if any.

The current work can be adopted on a commercial basis. For this purpose, we plan to develop a software library following the adapter design pattern. This library will support the methods of the current approach regarding the detection of bugs, fumigation and communication, by invoking the corresponding commands of the real precision agriculture system. In this way, smart communication protocols could be tested in the presented simulator and then transferred to the agriculture precision system. An enterprise could sell the precision agriculture hardware components with the corresponding software as a complex system. In the beginning, this system will include the smart neighbor protocol and the smart low-cost neighbor one. Later, when we develop new smart communication protocols that are more effective or use less power, farmers will have the possibility of updating their system by automatically incorporating these protocols. In addition, we will develop a mobile application to control this precision agriculture system from a tablet. This app will allow farmers to change the smart communication protocol and track the evolution of their agriculture fields. For example, it will show which areas currently have bugs, the average power used over time, the pesticide consumption and an estimation of the crop health based on the history of the presence of bugs. The commercialized complex system will include a low-cost tablet with this app pre-installed. One of the nodes of the WSN of the agricultural system will be connected to the Internet for uploading the data to a server. The farmer could control the system from their tablet connected to the server via the Internet. We will have to solve the problem of collecting the information from all the sensors with a low need of power. For example, sensors could send this information through the WSN to the central node with an Internet connection, and this could upload the information to the server. Sensors will probably only send information when they change their states. The server will update this information for consistently presenting all the available information when requested by the farmer.

## Figures and Tables

**Table 1:** Input parameter values of the experiments.

Input Parameter	Value
Initial number of affected areas	2
Columns of precision agriculture sensors	10
Rows of precision agriculture sensors	16
Ecosystem probability of increasing bugs per hour	0.10
Duration of simulation (hours)	48

**Table 2:** Results of the three communication protocols considering the means and SDs of 100 simulation executions for each one.

	Power (kW)	Crop Health (%)	Alive Bugs (%)	Pesticide (mL)
Broadcast	144.64 (24.88)	99.77 (0.07)	2.09 (6.56)	888 (365.23)
Neighbor	88.81 (4.28)	99.75 (0.10)	3.83 (8.60)	63.14 (25.03)
Low-cost Neighbor	67.64 (2.96)	99.20 (0.36)	6.13 (10.46)	74.77 (26.48)

**Table 3:** Robust tests of equality of means for comparing the three strategies.

		Statistic *a*	df1	df2	Sig.
Power	Welch	1216.406	2	170.104	0.000 **
	Brown–Forsythe	734.968	2	107.748	0.000 **
Crop health	Welch	119.561	2	173.960	0.000 **
	Brown–Forsythe	215.668	2	122.570	0.000 **
Alive bugs	Welch	5.498	2	190.944	0.005 *
	Brown–Forsythe	5.436	2	263.117	0.005 *
Pesticide consumption	Welch	255.165	2	176.104	0.000 **
	Brown–Forsythe	498.034	2	100.975	0.000 **

*^a^* Asymptotically F distributed; * statistically significant with a significance level of 0.01; ** statistically significant with a significance level of 0.001; Sig. denotes significance (*p*-value).

**Table 4:** Robust tests of the equality of means for comparing neighbor and low-cost neighbor strategies.

		Statistic *a*	df1	df2	Sig.
Power	Welch	1654.441	1	175.990	0.000 **
	Brown–Forsythe	1654.441	1	175.990	0.000 **
Crop health	Welch	215.190	1	113.940	0.000 **
	Brown–Forsythe	215.190	1	113.940	0.000 **
Alive bugs	Welch	2.889	1	190.884	0.091
	Brown–Forsythe	2.889	1	190.884	0.091
Pesticide consumption	Welch	10.190	1	197.375	0.002 *
	Brown–Forsythe	10.190	1	197.375	0.002 *

*^a^* Asymptotically F distributed; * statistically significant with a significance level of 0.01; ** statistically significant with a significance level of 0.001.

**Table 5:** Effect sizes between neighbor and low-cost neighbor strategies.

	Power (kW)	Crop Health (%)	Alive Bugs (%)	Pesticide (mL)
Mean difference	−21.18	−0.54	2.30	11.63
Cohen’s d	−5.75	−2.07	0.24	0.45

## Abbreviations

This manuscript uses the following abbreviations:

ABS	Agent-based simulator
ABS-SmartComAgri	The presented ABS of smart communication protocols in wireless sensor networks for debugging in precision agriculture
BGA	Binary genetic algorithm
BPSO	Binary particle swarm optimization
BQIGSA	Binary quantum-inspired gravitational search algorithm
CV	Computer vision
IoT	Internet of Things
PEABS	Process for developing efficient agent-based simulators
MAS	Multi-agent system
ODD	Overview, design concepts and details
QQIGSA	Quadrivalent quantum-inspired gravitational search algorithm
SD	Standard deviation
SEAD	Spatially Explicit Agricultural Dynamics
SMART	Scan-based movement-assisted sensor deployment method
TABSAOND	Technique for developing ABS apps and online tools with nondeterministic decisions
UAV	Unmanned aerial vehicle
UI	User interface
VANET	Vehicular ad hoc network
WSN	Wireless sensor network
